# Filter-Passing Virus in Disease

**Published:** 1919-02-08

**Authors:** 


### A LANDMARK IN MEDICAL RESEARCH?

### Filter-Passing Virus in Disease.

THE first information concerning a particularly interesting series of researches appears in the *British Medical Journal* of February 1. In this preliminary report it is stated that the causes of trench fever, trench nephritis and influenza have been definitely determined, and that an organism has in each case been isolated, examined, cultivated and re-inoculated to produce the same disease in animals. If these records will stand the searching test to which they must be put, and the methods employed are found possible of successful repetition, there is no doubt that we shall have produced one of the landmarks in medical research.

The work has not been limited to the war diseases alone, for experiment has extended to typhus, measles, mumps, and a few other common infections, the exact causes of which have so long baffled the profession. In each instance a considerable measure of success is claimed, and inspection of this forerunner of the detailed report at once suggests the possibility of a discovery, far-reaching in both its effects and application.

The whole is an investigation and report presented to the Director-General of Army Medical Services in France by Major-General Sir John Rose Bradford, A.M.S., Captain E. F. Bashford, R.A.M.C., and Captain J. A. Wilson, R.A.M.C. and its full publication will be awaited anxiously.

It has been known for some time that many diseases are transmitted by a very definite filter-passing virus found in the body fluids of the infected people, and endless animal inoculation experiments have been made. In fact, it has been possible to handle the infective agent in solution, as one might say, without ever being able to isolate a living cellular body or organism which was the producer of the toxin itself.

The enormous importance of these new discoveries lies in their having given the bacteriologist a definite living body which he can stain, see, recognise, and cultivate. The presence of such bodies has been recognised for a considerable number of years, but the fact that they could never be isolated has greatly hindered the possible experimental work required to find individual conditions necessary to develop efficient vaccines, anti-toxins, and the like.

So many times have we been disappointed in these minute organisms, so often the critic has so nearly proved his theory of "artifact" for the minute globules that it is impossible not to feel some hesitation in proclaiming that the problem has now been definitely settled. Still the preliminary report rings true with confidence, and it is to be hoped that the bacteriologist of to-morrow will find immense fields opened to him by the efforts and skill of these medical officers. We await their final report in the next issue of the "Quarterly Journal of Medicine."

